# A culturally informed model to enhance breast, cervical, and colorectal cancer screenings: perspectives of American Indian adults and healthcare providers in rural New Mexico

**DOI:** 10.1007/s10552-023-01721-y

**Published:** 2023-06-06

**Authors:** Shiraz I. Mishra, Prajakta Adsul, Samantha Leekity, Joseph Rodman, Andrew L. Sussman, Keith Kelly, Judith Sheche, Thomas Faber, Vallabh Shah

**Affiliations:** 1 Departments of Pediatrics and Family and Community Medicine, and University of New Mexico Comprehensive Cancer Center, University of New Mexico Health Sciences Center, 1 University of New Mexico, MSC 07 4025, Albuquerque, NM 87131 USA; 2 Department of Internal Medicine and University of New Mexico Comprehensive Cancer Center, University of New Mexico Health Sciences Center, 1 University of New Mexico, MSC 07 4025, Albuquerque, NM 87131 USA; 3 University of New Mexico Comprehensive Cancer Center, University of New Mexico Health Sciences Center, 1 University of New Mexico, MSC 07 4025, Albuquerque, NM 87131 USA; 4 Department of Family and Community Medicine and University of New Mexico Comprehensive Cancer Center, University of New Mexico Health Sciences Center, 1 University of New Mexico, MSC 09 5040, Albuquerque, NM 87131 USA; 5 Albuquerque Area Indian Health Service, 4101 Indian School Rd, NE, Albuquerque, NM 87110 USA

**Keywords:** Breast neoplasm, Colorectal neoplasm, Cervical neoplasm, Cancer screening, Cancer screening test, Health behavior, Health equity, American Indians, Community-based participatory research, New Mexico

## Abstract

**Purpose:**

American Indian/Alaska Native (AI/AN) populations have some of the lowest cancer screening rates compared to other racial/ethnic populations. Using community-based participatory research methods, we sought to characterize knowledge, attitudes, beliefs, and approaches to enhance breast, colorectal, and cervical cancer screening.

**Methods:**

We conducted 12 focus groups between October 2018 and September 2019 with 96 eligible AI adults and healthcare providers, recruited using non-probability purposive sampling methods from the Zuni Pueblo in rural New Mexico. We used the Multi-level Health Outcomes Framework (MHOF) to conduct a qualitative content analysis identifying mutable systems- and individual- level constructs important for behavior change that we crosslinked with the Community Preventive Services Task Force (CPSTF) recommended evidence-based interventions (EBIs) or approaches.

**Results:**

Salient systems-level factors that limited uptake of cancer screenings included inflexible clinic hours, transportation barriers, no on-demand service and reminder systems, and brief doctor–patient encounters. Individual-level barriers included variable cancer-specific knowledge that translated into fatalistic beliefs, fear, and denial. Interventions to enhance community demand and access for screening should include one-on-one and group education, small media, mailed screening tests, and home visitations by public health nurses. Interventions to enhance provider delivery of screening services should include translation and case management services.

**Conclusions:**

The MHOF constructs crosslinked with CPSTF recommended EBIs or approaches provided a unique perspective to frame barriers and promoters of screening utilization and insights for intervention development. Findings inform the development of culturally tailored, theoretically informed, multi-component interventions concordant with CPSTF recommended EBIs or approaches aimed at improving cancer screening.

## Introduction

Over the past few decades, the United States has witnessed a steady decline in overall cancer incidence (especially for men) and mortality, and an improvement in 5-year relative survival [1]. These gains, however, have not accrued evenly across populations. American Indian/Alaska Native (AI/AN) populations compared with non-Hispanic Whites (NHWs) have experienced persistent disparities in cancer incidence, mortality, and survival [2, 3]. Cancer disparities among the AI population in New Mexico (NM) are equally concerning (Note: when referring to NM-specific data, we use the abbreviation “AI” referring to American Indians only and not “AN” which includes Alaska Natives). Between 2012 and 2016, compared with the state’s NHW population, AI females had higher cervical and colorectal cancer incidence and higher cervical cancer mortality [4], while AI males had higher colorectal cancer incidence and mortality [4]. The state’s AI population was also more likely to receive diagnosis at a later stage (i.e., regional or distant) for all three cancers [5].

AI/AN populations have some of the lowest screening rates for the three screen-detectable (i.e., breast, colon-rectum, cervical) cancers compared to other racial/ethnic populations. In NM, the AI population in the Indian Health Service (IHS) Albuquerque Area compared with the state’s NHW population has substantially lower screening for breast (38.7% vs. 70.0%) [6, 7], colorectal (31.2% vs. 69.2%) [6, 8], and cervical (43.3% vs. 77.8%) [6, 9] cancers. AI/AN populations experience substantial individual-, and provider-/system-level barriers accessing screening services. These barriers include historical mistrust about healthcare services and institutions [10–12]; discordant patient–provider gender roles [11–13]; hesitancy to discuss cancer [14, 15]; privacy and confidentiality concerns [13, 16]; perceptions of not receiving quality and competent care [17]; fatalistic views about cancer [15, 18]; and no cultural or social norms for screening [19]. Further, Tribes and the IHS possess limited healthcare resources to address cancer disparities as healthcare is severely underfunded, services are often fragmented, and acute care needs take precedence over preventive care [20, 21].

Persistent cancer health disparities coupled with barriers to access and delivery of cancer screening for AI/AN populations argue for tailored, theoretically informed, multicomponent evidence-based interventions (EBIs) or approaches to enhance screening. The Community Preventive Services Task Force (CPSTF) has recommended EBIs or approaches to promote breast [22, 23], colorectal [22, 24], and cervical [22, 25] cancer screening. EBIs or approaches with “strong evidence” for effectiveness to enhance screening for these three cancers include multicomponent interventions, interventions engaging community health workers or navigators, client-oriented interventions (e.g., incentives, one-on-one education, and small media), and provider-oriented interventions (e.g., assessment and feedback, and reminder and recall systems).

With one exception [15], none of the research reviewed above on cancer and cancer screening perspectives was conducted among NM’s AI population. The diversity of AI/AN populations across the US, including 573 federally recognized Tribes [26] speaking about 150 Tribal languages [27], provides a compelling rationale to elicit representatively diverse perspectives from a broad range of AI/AN populations. The purpose of this qualitative inquiry that used a community-based participatory research (CBPR) approach was three-fold. First, using qualitative methods, elicit perspectives on knowledge, attitudes, and beliefs regarding the prevention and control of the three screen-detectable cancers among the AI population residing in the Zuni Pueblo in rural NM and among healthcare providers practicing at the Zuni IHS Comprehensive Health Center (hereafter, “Health Center”). Second, theoretically ground these perspectives with constructs of the Multi-level Health Outcomes Framework (MHOF) [28–30]. Identification of mutable individual- and system-level factors, such as knowledge, attitudes, beliefs, structural barriers, provider reminders and recall systems, and navigation services are particularly important as they represent potential targets for intervention. Third, map the perspectives and MHOF constructs on to the CPSTF recommended EBIs or approaches for potential multicomponent interventions.

## Material and methods

### Study setting and sample

We conducted the research at the UNM Health Sciences Center (HSC) satellite office and the Health Center, located on the Zuni Pueblo. The UNM HSC’s Human Research Review Committee and the Southwest Tribal Institutional Review Board approved all aspects of the research protocol. Eligibility criteria included adults (21–75 years) who self-identified as tribal members and resided in the Pueblo. Consistent with principles of qualitative research methodology, we used non-probability (non-random) purposive sampling techniques [31] to identify and invite eligible community members and healthcare providers for the focus groups. We identified community members based on recommendations from the Tribal Advisory Panel (TAP) and word of mouth, and recruited them based on selected characteristics (i.e., age, gender). Further, TF used the same sampling technique to invite healthcare providers practicing at the Health Center to the focus group. These healthcare providers included those who expressed an interest in the study topic, were primary care providers, and did not have clinical or administrative responsibilities at the time of the focus group.

The Health Center is the only healthcare facility located on the Zuni Pueblo. It provides standard cancer screenings on a routine basis to all age-eligible patients being seen for primary care. These include digital mammogram for breast cancer (ultrasound for diagnostic breast exams), Pap smear for cervical cancer, and immunochemical fecal occult blood testing or colonoscopy referral (based on patient preference) for colorectal cancers. The Health Center does not offer mammography mobile vans, self-sampling for cervical cancer screening, and colonoscopies. Patients are referred over two hours away to Albuquerque for colonoscopies, with wait times for scheduling them often six to nine months. It is open 24/7, has evening primary care clinic hours, provides option to the patients to choose gender concordant providers, and has an appointment reminder system.

### Theoretical framework

The MHOF [28–30] informed the discussion guide and analysis approach. It has been used to study antecedents of health behavior in diverse cancer control studies [29, 30, 32–38]. We elicited participants’ perspectives on several modifiable individual- and system-level MHOF constructs with a view of integrating these constructs in cognitive-behavioral interventions. The MHOF originates outside the cultural context of Zuni and serves as a guiding framework that will be adapted and revised based on emerging data.

### Community-based participatory research

We employed engagement processes founded on CBPR principles, which envision researchers and community members establishing a non-hierarchical partnership for all phases of the research [39–41]. CBPR is a necessary methodology for research with AI/AN populations [42–45] as it addresses mistrust of research and researchers. Zuni project staff and students provided critical input on all aspects of the research. Tribal leaders, recruited to the Tribal Advisory Panel (TAP), shared their expertise on development of the focus group guide, participant recruitment strategies, and interpretation of findings. We tailored protocols for cultural acceptability and respectfulness, and built sustainable processes for long term impact of the project.

### Focus group procedures

We convened three clusters of eligible AI community members, stratified by age and gender. These clusters included: females ages 21–49 years for discussions on cervical cancer and HPV, females ages 50–75 years for discussions on breast, colorectal, and cervical cancers, and HPV, and males ages 50–75 years for discussions on colorectal and prostate cancers. In this report, for men 50–75 years, we only analyze and present focus group discussions on colorectal cancer. Zuni research assistants (SL, TB, KH, KB) or the first author moderated the discussions with community members. ALS moderated the discussions with healthcare providers. The discussions were primarily in English, although there were instances when community members spontaneously conversed in the Zuni language, “Ashiwi awan benawe.” We ended data collection after reaching thematic saturation [46, 47]. At the end of each discussion, the first author addressed misconceptions raised during the discussions. Participants completed a socio-demographic survey prior to the discussion. They received merchandize cards based on the time commitment of their respective groups. Men and women ages 50–75 years received $75 merchandize cards for groups that averaged about 90 min; women ages 21–49 years and the providers received $50 merchandize cards for groups that averaged about 60 min.

We conducted 12 focus groups [48] between October 2018 and September 2019, with each group comprising 4–13 participants. The moderator followed a discussion guide (available upon request) specific to the community member’s age/gender strata and the healthcare providers. The guides explored individual- and systems-level facilitators and barriers to cancer screening, and strategies that can enhance screening behavior. For the community member focus groups, the key areas of inquiry included knowledge about the specific cancer (i.e., risk factors, prevention); perceptions of susceptibility to and severity of cancer; personal and family history of cancer; knowledge about and use of cancer-specific screening test; social support and social norms for cancer screening; communications with healthcare providers regarding cancer and cancer screening; cultural and traditional beliefs regarding cancer; and strategies to enhance screening. For the healthcare provider focus group, the key areas of inquiry included their perceptions of the community’s beliefs and attitudes regarding preventive behaviors and cancer prevention, help-seeking behavior, and knowledge about cancer; providers’ approaches and experiences with counseling patients about cancer screening; challenges to providing cancer screening; and potential strategies to improve cancer screening in the community. Stem questions emphasized a strengths-based approach [49, 50] for identifying solutions to barriers and potential health-promoting intervention strategies. The purposive sampling technique employed in addition to iterative data collection and analysis, leading to data saturation, generate confidence that the findings of the research will serve as the basis for testable interventions. Moreover, through systematic and rigorous application of qualitative methods, findings of this research will be conceptually and culturally relevant.

### Data analysis

Pairs of research assistants, conversant in both English and “Ashiwi awan benawe,” transcribed verbatim the audio recording and verified the transcription. Transcripts and the note-taker’s observations served as the primary data sources. We employed a sequential approach to our analysis, importing transcripts, coding, and querying using NVivo 10, a qualitative data analysis software program. First, we used a systematic iterative process to code transcripts for the MHOF constructs to ground the participants’ perspectives within the theoretical framework. In all, we coded the transcripts for 16 MHOF constructs, besides potential intervention strategies discussed by the participants. We then identified preliminary and emergent themes of importance for each MHOF construct. Several team members independently read each transcript to identify preliminary findings, which facilitated confirmation of emergent themes. The initial analysis resulted in a coding template consisting of 40–53 themes and subthemes depending on the age/gender or provider cluster. After coding all transcripts, we queried the database by coding categories for more refined interpretive analyses. Next, we cross-linked the emergent themes to the CPSTF EBIs or approaches to ground the analyses in actionable EBIs or approaches backed by “strong evidence” for effectiveness to enhance screening for the cancers under study. Lastly, we presented our preliminary findings to the TAP and Zuni Tribal Council members for their interpretation of the findings and data narratives. We analyzed the quantitative survey data to produce descriptive assessments of the participants’ demographics.

## Results

Table 1 presents socio-demographic characteristics on 85 community members and 11 healthcare providers who participated in the focus groups. The majority of community members were Zuni tribal members (with three participants having non-Zuni tribal affiliation), spoke “Ashiwi awan benawe” either often or always, were mostly single, high school graduates or college educated, in the workforce, and had health insurance. Although IHS does not provide health insurance, nearly one-quarter of the community members indicated IHS as their health insurance. This interpretation may be due to community members’ reliance on IHS for all their health care and costs. The majority of healthcare providers were young [30–39 years], female, NHW persons, specialized in family medicine, and had been in medical practice for less than 5 years.

**Table 1:** Socio-demographic Characteristics of Community Members (n = 85) and Healthcare Providers (n = 11) Participating in the Focus Groups

	Members (*n* = 85)		Providers (*n* = 11)	
	*n*	%	*n*	%
*Age, in years*				
20–29	5	6.1		
30–39	11	13.4	7	63.6
40–49	11	13.4	2	18.2
50–59	29	35.4	1	9.1
60–69	15	18.3	1	9.1
70 or older	11	13.4		
*Sex*				
Male	30	35.3	5	45.0
Female	55	64.7	6	55.0
*Race/ethnicity*				
American Indian	82	96.5	1	9.1
Non-Hispanic White			10	90.1
Other	3	3.5		
*Tribal affiliation*				
Zuni	82	96.5		
Non-Zuni	3	3.5		
*Speak Ashiwi awan benawe (Zuni language)*				
Always	34	41.0		
Often	31	37.4		
Seldom	16	19.3		
Never	2	2.4		
*Marital status*				
Married or living together	31	37.4		
Single (divorced, widowed, separated, never married)	52	62.7		
*Education level*				
Some high school or less	19	22.4		
High school graduate	28	32.9		
Some college	36	42.4		
College graduate	2	2.4	11	100.0
*Employment status*				
Not in the workforce (retired, student, homemaker, unable to work, not seeking work)	19	23.5		
In the workforce (employed, self-employed, unemployed)	62	76.5	11	100.0
*Medical specialty*				
Family medicine			9	81.8
Internal medicine			2	18.2
*Years in medical practice*				
< 5			6	54.5
5–10			2	18.2
> 10			3	27.3
Health insurance status*				
Private	3	3.5		
Medicare	19	22.4		
Medicaid	63	74.1		
Other	5	5.9		
No health insurance	2	2.4		
Received care through the Indian Health Service	20	23.5		

Percentages are of valid responses. Not all total equal 100% because of rounding error

^*^ Participants could provide multiple responses

We have organized the results in two sections. Section I links community member and provider perspectives with selected MHOF constructs, and Section II, building on the strengths-based approach, links community members’ suggested interventions to enhance screening with the CPSTF recommended EBIs or approaches.

I. Community Member and Provider Perspectives Linked with Selected MHOF Constructs

We organized results by three MHOF constructs: knowledge, barriers and supports (e.g., community member, provider, system, society), and communications (including communication with providers and with others). We have included exemplary quotes below and Table 2 presents additional quotes supporting each construct.

1. Knowledge

**Table 2:** Community Member and Provider Perspectives Associated with the MHOF Constructs

Construct	Community member quotes	Provider quotes
Knowledge	C1 “We do need to know [more] about cervical cancer because…I myself am guilty of not going to the hospital to get regular pap smears…” (Women 21–49) C2 “People know about breast cancer, but these other two [cervical and colorectal cancers], you know, we don’t really know about.” (Women 50–75) C3 “I don't know how it is. Is it a germ? I don't know what cancer is.” (Women 50–75) C4 “Down here they said it [cancer] comes from our food, with that posole, our bread, lot of grease, and all those. That’s how they said it comes we get sick with that right away like diabetic, cancer, whatever, kidney failure. Yeah that’s what they said causes that, cause were eating too much of that.” (Women 50–75) C5 “See that’s why I’m asking. Is it in the air? Is it in our water? Is it the food? Is it where we live? What is it? Where is it? That was my first question.” (Men 50–75) C6 “It’s heredity. If you’re great grandparents had it, cancer, […] it’s not passed down to the first one it’s passed down to the second or third, cause […] my mon and brother had cancer. My mother had cancer of the pancreas and my uncle had stomach cancer or colon cancer and none of us received it […] and then my daughter had […] cancer of the uterus…” [Women 50–75] C7 “As soon as I heard about it [HPV vaccination] and when my daughter started going into her teen years and once the hospital started doing that I already had her go and you know get her shots for that…” (Women 21–49)	P1 “… now a lot of people can come in and are like, ‘Do I have cancer? […] Check me out,’ […] [they] don’t think of it as colon cancer, breast cancer, cervical [cancer] […] these are all different things. I don’t think that’s […] the way people think of it […] there’s a way that we should be able to figure out if someone has cancer…” P2 “I think for me, my patients, if they have a family member who have had something and have had to help them deal with it they are more incline[d] to get checked. I have a patient who just recently died from rectal cancer and her younger sister who is less than 50 asked for a colonoscopy, so I think… and that’s just one but, but, yeah I think […] when they see what happens to them.”
Barriers & supports (community member, provider, system, societal)	C8 “Frustration for me because every time I go up there [to the community clinic] they say…there’s nothing wrong me, but I come back. So I don’t know if it’s like they only pick and choose who they want to help. I don’t know, I’m not sure.” (Women 50–75) C9 “It’s kind of scary…to find out.” (Men 50–75) C10 “[It’s] probably [because of] embarrassment. I mean, who wants to go drop their pants…who wants to put their stool on a piece of paper and…send it to the doctor?” (Men 50–75) C11 “I would recommend you […] make sure that you’re informed and of course family’s always involved […] my family, everybody […] we’re open to […] anything cause […] that’s part of being a family […] to care for another […] I first inform[ed] my spouse and […] when I was through my procedure […] I had to tell them [so they] knew what’s going on with the procedure […] with family it’s always good cause they care and […] everybody wants to be healthy …” (Men 50–75) C12 “My brothers and uncles we’ve all got tested and they always keep telling me or asking me if I got tested and I always tell them not yet and they say get your butt in gear and get yourself tested it’s nothing to be embarrassed about” (Men 50–75)	P3 “…a lot of women…don’t show up following…an abnormal Pap or repeat Pap, and I think that it’s a lot of being scared for mammograms…people are scared and they don’t want to know the results and they think the worst…] P4 “[It’s] fear, maybe jinxing, like you’re fulfilling it if you go looking for it, whereas if you didn’t look for it, it wouldn’t happen…” P5 “I honestly feel like the test to my patients and the community members I’ve talked to, seem irrelevant, unfortunately. It seems beside the point…I think there’s a certain fatalism, this isn’t by any means a 100 percent or 80 percent, but I do hear that sometimes, when people feel like, ‘what’s going to happen [is] what’s going to happen.’” P6 “…colonoscopy is really important but is an abstract idea when you are having issues with financial insecurity, you’re having issues with the child care, you’re having this stress at home, you know, whether it’s intimate partner violence or kind of like everything, colonoscopy is the least important, Pap smear is the least important, all that, it’s like, you know it’s important, they articulate that, but there’s just so much other stuff.” P7 “… in [Facility Name] for whatever reason them not showing up to appointments […] I think appointments are difficult […] especially […] for a colonoscopy […] there’s a lot of systematic problems […] waitlist, wait times, and contract health referring delays, and transportation […] gas money is always […] the reason.” P8 “I wish that we could give mammograms […] on demand…” P9 “Transportation is the issue for patients, because usually when you go for a screening colonoscopy you have to take someone […] so just to have transportation to get there, to the place, you’re going to have someone to go with you. So if somebody else […] maybe takes a day off from work […], taking care of kids to be able to do this, I mean, I’m always getting issues about transportation.” P10 “That’s also a tricky thing, of finding a good way to document [family history of cancer] in the EHR [electronic health record] so it carries forward […] so other people can find that information […]” P11 “[…] I think that the colon cancer screening […] I think we really struggle there […] it’s like we give kits to people but ‘you didn’t return your kit.’ ‘Oh, I still have it at home. I’ll bring in that’.”
Communication	C13 “Yes, I would talk to my spouse about it [colonoscopy]…she went through it and told me what to expect […] I’m willing to go through it […] I’m willing to tell other people about it. Certainly some of my friends that I went to school, some of them are already long gone […] so I don’t want to be like them. I want to be informative on the situation when this type of disease comes up.” (Men 50–75) C14 “…we all share laughter, we all share humor so it would maybe put women more at ease […] so I, I would say you know just keep it light hearted […] at least laugh about it but then at the same time you know be serious about it [and if get] yourself checked it would definitely help” (Women 21–49 C15 “Whatever my doctor tells me I’m […] very confident that he’s going to help me […] with my health issues. So, yes, I would take tests [recommended by my provider]” (Men 50–75) C16 “I like going to my appointment cause I’m comfortable with my female doctor and just do whatever she tells me to do” (Woman 50–75) C17 “Always ask for a female. If not, schedule it another day where the female is working.” (Women 21–49) C18 “It’s very scary. In our traditional ways we don’t talk about death. It’s taboo, so we tend to just keep things to ourselves. We don’t think about getting life insurance because its taboo. We have…relatives that are on the verge of death; we don’t want to talk about it because what you say is what you get and maybe somebody will point the finger at you saying, ‘Why did you say that?’” (Women 50–75) C19 “It’s probably not something that people really want to talk about…I mean, cervical cancer, that’s pretty…that’s almost as private and personal as a person can get, you know, cause it’s inside of us and…it affects…your womanhood…if it’s cervical cancer then…it can hurt your chances of…[having] kids or…just being a being a mother…” (Women 21–49) C20 “This conversation [about cancer and cancer testing] don't come up…we don't talk about it, you know…cause we're guys, you know.” (Men 50–75) C21 “Now a days these doctors, my doctor now, just wants to see [me] for a quick minute. They’ll let you wait for a long time and then he comes in for like maybe two minutes and that’s it. [Laughs].” (Men 50–75) C22 “To me [talking to the doctor about cancer screening] is hard. It’s hard because the long terminology words; whatever they’re using. I mean, they don’t have the time to explain it to you in the terms that you would understand and it’s kind of difficult to say, ‘okay.’ Or, if I say no, what am I gonna put myself through or what am I gonna end up having later. So it’s kind of difficult…” (Women 50–75)	P12 “We talk about it [cancer] all the time and every time she’s able to articulate why she needs a colonoscopy but […] her work […] it’s just more important right now […]” P13 “When I first came here I think I put more effort, energy in trying to talk people into screening colonoscopy […] but maybe because it’s a better test and a lot of people would like sort of sit and be like we explained it and then they’ll be like, ‘No, no, no, I just want to do the FOB card’, and then maybe we can try it, maybe not, and then among people that I did talk to […] they would often cancel or no show” P14 “‘I don’t [know] what type.’ ‘Can you like ask your mom like what surgery she had,’ ‘Like no, no we don’t talk about it.’ I hear that a lot, like, ‘We don’t ask those private questions.’ P15 “It’s just good to talk in the third person…when I first started working here…I don’t know who told me that it’s better if you suggest [to patients] without saying… ‘this is to make sure you don’t have colon cancer’ [instead say]… ‘this is to make sure everything’s healthy inside,’ …so try [to] use more gentle phrases. Try to avoid the word [cancer] when talking about cancer if it feels like too personal…” P16 “In the last year the women who had Pap smear, [I would ask them], like, ‘hey why are you having a Pap? […] Do you know what a Pap smear is for?’ and twenty percent know that it’s for cancer; most people are just like ‘to make sure everything’s okay down there.’” P17 “For the stool test [I] joke with my patients. You know, I say, ‘Oh look, we gave you a little gift today.’ And I…say, ‘You know, look, I know nobody really wants to do these but this is why, you know, this is why we do [this].’”

Across the groups, community members consistently reported variable knowledge and awareness regarding cancer etiology, specific cancer types, and associated screening tests. Community members cited a need for clear information about different cancers, screening tests, and prevention strategies (Table 2, quotes C1-C2). In response to questions about potential causes of cancer, community members expressed a range of views pertaining to causal possibilities, including environmental contributors (e.g., airborne contaminants and water), food sources attributable to dietary changes over time, and heredity (Table 2, quotes C3-C6).

When aware or informed, community members reported receptivity to preventive strategies such as HPV vaccinations and screening (Table 2, quote C7).

“Screening is really important. So that way at least there [will] hopefully [be] some […] intervention, prevention awareness that most people will start understanding.” (Men 50-75)

Providers generally concurred, noting that while community members maintained a basic knowledge of cancer, they attributed low screening rates to individual- and system-level barriers discussed below under barriers and supports. Providers indicated that community members wanted to know about cancer and screening, especially if they had to help or take care of a family member who had cancer or had a family history (Table 2, quotes P1-P2). As a provider noted:

“I think there are some patients who definitely want to know if they have cancer. I think people who have family history, whether its siblings, parents are kind of more interested because they worry that it could be affecting them.”

2. Barriers and Supports

Community members cited systemic barriers giving rise to frustration (Table 2, quote C8), fear of cancer diagnosis (Table 2, quote C9), and embarrassment given test procedures (e.g., Pap test, stool test) (Table 2, quote C10) and concerns pertaining to confidentiality given the likelihood of potential interactions with medical staff who may be neighbors or family members.

“For me it…was really the embarrassment because there’s a lot of [local] nurses now and I don’t want another [local resident] looking at me down there.” (Women 21–49)

“Well…it’s confidentiality. That’s what I was worried about. Our people are nosey. When you walk into the hospital you’ll get stared at hard.” (Men 50–75)

Community members also identified the benefits of supportive elements (i.e., family members) to promote better health and overcome screening hesitancy and embarrassment (Table 2, quotes C11-C12).

Providers echoed interrelated concepts of fear/denial, fatalism, and procrastination as barriers to cancer screening (Table 2, quotes P3-P5).

“It’s on people’s radars…it’s on the back of their minds, but [there is] a very strong denial…”

Providers described social determinants of health as important barriers. Further, providers reflected on competing priorities (i.e., financial insecurity, child care, stress) among their patients given social conditions of poverty and other demands (Table 2, quote P6).

Providers mentioned additional systems-level barriers that precluded access to timely screenings. These included transportation issues, long wait times at clinics, inflexible clinic hours, inability to provide screening on-demand, lack of child care, lack of reminder systems (for patients and providers), limitations of the Electronic Health Record (EHR) system, and no system or procedures in place to collect completed stool kits (Table 2, quotes P7-P11).

3. Communication

We explored levels of communication to discern the degree to which one’s social network influenced perspectives on screening behaviors as well as self-reported interactions with healthcare providers. With regard to social networks, community members indicated the importance and need for discussing health behaviors and cancer screening with family members (Table 2, quote C13):

“… whatever you’re told [from the doctor about being diagnosed with cancer], you know, I would go home and discuss it with my family to let them know what’s going [on]…” (Women 50–75)

“…in order to break the cycle you have to sit and do not be afraid, not be ashamed, not be embarrassed, but to seriously talk about it.” (Women 21–49)

Community members expressed that the use of humor, confidence and trust in the provider, and gender concordance with their provider eased communications of serious issues (Table 2, quotes C14-C17). Community members, however, attributed a lack of conversation about topics such as cancer, cancer screening, and death to generalized cultural traditions, gender norms, and privacy concerns (Table 2, quotes C18-C20). Additionally, systems-level factors precluded expanded discussions on healthcare needs. These factors included brief clinic visits and use of medical jargon and, which may be a function of insufficient clinic manpower or lack of prioritization of cancer screening at the clinic (Table 2, quotes C21-C22).

Providers emphasized prioritizing communication with patients about cancer screening as they mostly attributed low screening rates to patient choices and lack of follow-through. They noted in their patients an awareness about and desire to act on the need for screening but not going through it (Table 2, quotes P12-P13). A provider also noted the cultural taboo limiting discussion about cancer within family contexts (Table 2, quote P14).

Providers indicated that they utilize culturally tailored counseling strategies in recognition of the need to address cancer screening in an indirect manner (i.e., communicating in third person) (Table 2, quote P15), a strategy also employed by their patients (Table 2, quote P16). Providers also use humor to communicate the importance of the test (Table 2, quote P17).

II. Building on the Strengths-Based Approach, Linking Community Members’ Suggested Interventions to Enhance Screening with The CPSTF Recommended EBIs or Approaches

Building on the strengths-based approach, we cross-referenced major thematic findings derived from community member and provider focus groups with the CPSTF recommended EBIs or approaches to guide identification of interventions or approaches aligned with community perspectives (Table 3). We limited our analyses to CPSFT recommended EBIs or approaches that have “strong evidence” in support of their effectiveness (previously described). Further, we aligned the interventions or approaches with the MHOF constructs presented in Table 2.

1. Potential Client-Oriented Interventions

**Table 3:** Linking Community Members’ Suggested Intervention Strategies to Enhance Screening with the CPSTF Recommended EBIs or Approaches

Strategy	Type of intervention	Quote	MHOF construct
*Potential client-/patient- oriented interventions*^a^			
Increase demand for screening services	One-on-one education	1. “I would hope that they can have field health nurse and go out [to my brother’s home] and you know talk with him one and one. I’m pretty sure he would be open to doing that [FOBT]…you know, offer it to him; say, ‘you can do this on your own privacy…I’ll even come and pick it up…’” (Men 50–75)	Knowledge Communication with providers Barriers and Supports
		2. “I think that’s one way that would really help a lot: [a] field health nurse…I know…a while back they use to call the nurse on somebody who wouldn’t go [to the hospital]…say this person got this [problem or disease] and…they won’t go to the hospital or something wrong with their kid, their child…I remember they used to call the…field nurses cause they would go down and check on them…” (Men 50–75)	
		3. “…have like, the public health nurses just go to individuals who have not had a scheduled pap smear or HPV vaccination and go to those individuals and say you know this is what we’re doing why we’re doing it you know we encourage you to do this and then maybe then that’ll get them to do it.” (Women 21–49)	
	Group education	4. “I think [group sessions]…would be really [helpful], not only for this type [of cancer] but…cancer itself [more generally]…having something like this [group session] in order not [to] get…colon cancer…maybe one or two of us will go…or three or four…I think that will be a good thing…I’m getting the guts now [to talk about cancer].” (Men 50–75)	Knowledge Communication with others Communication with providers
		5. “…like this group session…after this…maybe it can be start from there…talking one-on-one with other family members and then maybe it can be a chain reaction from there…” (Men 50–75)	
		6. “Yeah I’m pretty sure [people would want to attend group sessions] cause…they will…be…interested in learning about…this illness, this sickness…especially when…family members…die, you know, they pass on it and I’m sure they’re willing to learn and know more about…what to do about it or [learn] what causes it.” (Men 50–75)	
	Client reminders	7. “You miss one appointment and then…they say that…since you missed you got to wait so long to get your next appointment.” (Men 50–75)	Communication with providers
	Small media	8. “…if there’s pamphlets or something like that…you could distribute them…at the stores or something like that. Or…make a big poster…I’m sure somebody would…have an interest in it.” (Men 50–75)	Knowledge
		9. “…give them out [print outs, pamphlets] to each household to read what cancer is all about, cause some people…they don’t like to get involved with other people. They keep to themselves, you know.” (Women 50–75)	
Increase community access	Reduce structural barriers	10. “No transportation maybe where their health facility is located or maybe they don’t go to that certain facility, hospital.” (Women 21–49)	Barriers and Supports Knowledge
		11. “A lot of people have difficulty getting rides to their appointments… I think that’s one thing that’s kind of hard for people to get to appointments, especially in Albuquerque or… when they’re scheduled at a different facility instead of here.” (Women 50–75)	
		12. “I prefer [Facility #1 Name]…because they just gave me [the] run-around over here [Facility #2 Name]. They…keep telling me ‘go here, go there’ and now I went through lot of my tests for my…sickness that I got. To this day they haven’t…sent my results to [Facility #1 Name] so I called and I asked them ‘where are my results?’ and they told me you have to have them send it over here at the hospital. They said I have to do like a walk-in just to talk about my results and I’m like why, why, why can’t they just…schedule me and tell me, ‘okay, come up, we’ll talk’ but they said [there was] like a month [wait] for [an] appointment over here…I asked the [Facility #2 Name] doctor and I said ‘when am I going to know my results?’ And that…doctor [said] that we’re going to send your results to [Facility #1 Name], so I figured they already did but nobody hasn’t said anything to me yet.” (Women 50–75)	
*Potential Provider-Oriented Interventions*^b^			
Increase provider delivery of screening services	Provider assessment and feedback	13. “To me [talking to the doctor about cancer screening] is hard. It’s hard because the long terminology words; whatever they’re using. I mean, they don’t have the time to explain it to you in the terms that you would understand and it’s kind of difficult to say, ‘okay.’ Or, if I say no, what am I going to put myself through or what am I going to end up having later. So it’s kind of difficult…” (Women 50–75)	Communication with providers Barriers and Supports
		14. “I also think it has to do with communication between the patient and the doctor you know sometimes the doctor will ask or you know sometimes it’s just like brushed aside with the patient or you know the patient not wanting to accept whatever they’re told…” (Women 21–49)	
		15. “Nowadays these doctors, my doctor now, just wants to see [me] for a quick minute. They’ll let you wait for a long time and then he comes in for like maybe two minutes and that’s it.” (Men 50–75)	

^a^CPSFT recommended EBIs or approaches that have “strong evidence” in support of their effectiveness

^b^Providers did not share any perspectives on “provider reminder and recall systems” (CPSTF recommended EBI or approach with “strong evidence”)

Community members endorsed a series of interventions or approaches to increase demand for screening services. They advocated one-on-one and group education interactions led by community health workers (i.e., field nurse, public health nurse, or other healthcare professional) to increase awareness and address knowledge gaps. They noted that offering both types of educational sessions ensures alignment with community member preferences. Further, they suggested systems-level approaches that addressed transportation barriers, such as having field health nurses provide education around screening to community members in their homes, offer to pick up stool test kits (Table 3; quotes 1–6).

“One-on-one [sessions would be best to encourage people to get screened], so that way they’ll be just there with the person and however they’re feeling they’ll do everything that is asked of them…without having to be afraid of the people that are sitting in there. Maybe if it’s crowded like this [focus group] they’ll be embarrassed and they won’t ask a lot of questions...” (Women 50-75)

In response to concerns expressed about healthcare services at facilities where they sought care, community members supported systems-level solutions such as a scheduling system or client reminders about appointments. In addition to serving a pragmatic purpose, reminders were also viewed as a sign that the healthcare facilities were invested in community members’ wellbeing. (Table 3; quote 7).

“…schedule appointments ahead of time and maybe people will make arrangements to keep their appointments…” (Women 50–75)

“I was supposed to go [to the urologist last year] but…I don’t know why they…rescheduled my appointment so I had to reschedule my appointment then and I haven’t gone [back].” (Men 50–75)

Use of small media, including pamphlets and flyers, were suggested by community members as an educational strategy. (Table 3; quotes 8–9) Lastly, to increase access to screening by reducing structural barriers, community members emphasized systems-level strategies such as flexible and responsive appointment scheduling and assistance, and as needed, overcoming transportation barriers. (Table 3; quotes 10–12).

2. ***Potential Provider-Oriented Interventions***

Community members noted challenges to provider communication or, in some situations, perceived lack of empathy (e.g., the provider not spending sufficient time with the patient or brushing aside the patient’s concerns). They also noted translation and case management services could improve doctor–patient communication. (Table 3; quotes 13–15).

“Sometimes…you don’t understand what the doctor is saying to you. It’s hard to understand, and you repeat it in your own way and then I don’t know…” (Men 50–75)

## Discussion

The goal for this CBPR-informed research conducted with AI community members and providers in rural NM was to identify salient and mutable MHOF constructs, crosslinking them with CPSTF recommended EBIs or approaches, to subsequently design and implement a multicomponent intervention to enhance cancer screening rates. We identified important systems-level barriers that limited community access to screening services at healthcare facilities the community members seek care (which may not be the Health Center located on the Zuni Pueblo). These included inflexible clinic hours, inadequate provider communications, lack of transportation, privacy/confidentiality concerns, lack of gender concordant provider, need for translation and case management services, and the need for patient and provider reminder systems.

Strategies to enhance community demand for and access to screening should include one-on-one and group education, use of small media, and interactions with and home visitations by community health workers. These educational strategies should focus on the specific cancer type and their screening modalities. Strategies to enhance provider delivery of screening services should include the provision of translation and case management services. Although cultural norms generally preclude discussion about cancer or medical history among family members and friends, there appears to be some effort in the Zuni community to transcend this taboo. Community members and providers cited several instances of open communications regarding cancer and screening tests among family members and with providers.

The findings corroborate those documented from qualitative studies among AI/AN populations across the US on cancer screening [10–18]. Similar to the findings reported here, those studies also documented hesitancy to discuss cancer and cancer screening, negative experiences with the healthcare system, transportation barriers, fear of screening results, lack of quality and competent care, and concerns about privacy and embarrassment. This research extends these findings by documenting important individual- and systems-level barriers and supports for screening. Individual-level factors to support screening included the willingness to act on information, a growing willingness to discuss cancer and cancer screening with family members, trust in healthcare providers, and receiving gender concordant care. Systems-level barriers encountered where community members sought healthcare services included frustration with healthcare providers, inflexible clinic hours and the need for on-demand service, need for translation and case management services, limitations of the EHR system, and no patient and provider reminder systems.

Community members identified several CPSTF recommended EBIs or approaches to enhance screening that would work well in the context of their community, cultural preferences, and healthcare environment. Potential client-oriented EBIs or approaches to improve community demand included enhancing knowledge through culturally appropriate one-on-one and group education; however, the rationale for each strategy was quite different. One-on-one education and interactions with public health nurses could ensure privacy, while home visitations and mailed screening tests (for colorectal and cervical cancers) would address the lack of transportation as a structural barrier. Group education was recommended to address knowledge as some community members felt empowered to leave behind their fears and taboos and openly discuss cancer and its implications in such a setting. Further, group education could lead to one-on-one discussions with family members. Small media would complement these educational strategies. Additional intervention strategies could include translation and case management services to address poor or brief doctor–patient communications and interactions. Providers highlighted that cultural taboos limited discussions around cancer within the family context. They circumvented this by utilizing culturally tailored communication strategies such as using gentler phrases such as colorectal “health” instead of colorectal “cancer” or talking in the third person (a strategy also employed by community members). Providers expressed the need to use humor when discussing important health-related topics. To the extent that the community members identified CPSTF recommended EBIs or approaches [22–25] that are also endorsed by other populations is indicative that similar interventions or approaches may be appropriate across broad populations. At the same time, a long history of research has underscored the importance of “cultural tailoring” of interventions to reflect the cultural context, norms, and mores of different populations [51–53]. This tailoring could be through inclusion of culturally appropriate language and communication strategies, art, testimonials, and role models.

The diversity of AI/AN populations argue for the importance and need to document these perspectives from AI/AN populations across the US to better inform indigenous narratives around health. The current study is unique for its contributions as one of the first studies in AI/AN populations to document and contrast cancer control and prevention perspectives elicited from community members and healthcare providers practicing in the same community, and to link these perspectives to modifiable constructs of the MHOF and CPSTF recommended EBIs or approaches for intervention development. The focus group format allowed us to discover the tension between the desire to take charge of one’s health and systems-level, cultural or affective reasons for not doing so.

Our study has strengths and limitations. A compelling strength of this study is the use of a theoretical framework (MHOF) to organize the qualitative data and link it with CPSTF recommended EBIs or approaches, thus making the data actionable in terms of potential multicomponent EBIs to enhance cancer screenings. Further, use of the MHOF has helped contextualize the findings within a larger body of literature. One of the MHOF’s construct is “cultural beliefs.” Given the population of interest for this research, the focus group guide included questions and probes to pursue this line of inquiry. However, cultural imperatives preclude talking about certain topics such as cancer, as documented in this study and elsewhere [15]. We recently documented no significant associations between health literacy/numeracy and cancer screening in the Zuni Pueblo, a finding not aligned with many studies [54]. We have postulated that “standard” questions used to assess health literacy may need to be tailored to the contextual and cultural realities of the populations under study. Another strength is implementation of the CBPR methodology that facilitated bi-directional partnerships with community members on all phases of the research. This engagement drives development of indigenous data narratives, commits to sustainability, and makes the research socially actionable to address the cancer control needs of the Zuni community. The findings from this qualitative study are based on one Tribal population and group of healthcare providers in one rural NM community. As such, they are not representative or fully generalizable to heterogeneous AI/AN populations across the US. The limited number of focus groups per age/gender clusters could further reduce generalizability of our findings. Despite the limited number of groups conducted, there was convergence on the main themes reported across the various clustered groups and we achieved thematic saturation on areas of inquiry. Given the breadth and depth of focus group discussions, what we may have lost in terms of generalizability based on the relatively small number of focus groups, we gained through contextually rich discussions.

In conclusion, the MHOF constructs linked with CPSTF recommended EBIs or approaches provided a unique perspective to frame barriers and promoters of screening uptake and insights to develop multicomponent EBIs. The insights gained from this qualitative research are being validated through a community-wide quantitative survey. These qualitative and quantitative data will be used for culturally tailored, theoretically informed, multicomponent interventions concordant with CPSTF recommended EBIs or approaches aimed at improving screenings for colorectal, breast, and cervical cancers among age-eligible men and women in the Zuni Pueblo in rural NM.

## Data Availability

The authors are committed to the open and timely dissemination of unique research outcomes in compliance with Tribal and federal (NIH) policies. The authors will share data being cognizant of the data sharing needs and goals of the participating Tribe, and are guided by the Tribal data sharing agreements.
